# Biological data annotation via a human-augmenting AI-based labeling system

**DOI:** 10.1038/s41746-021-00520-6

**Published:** 2021-10-07

**Authors:** Douwe van der Wal, Iny Jhun, Israa Laklouk, Jeff Nirschl, Lara Richer, Rebecca Rojansky, Talent Theparee, Joshua Wheeler, Jörg Sander, Felix Feng, Osama Mohamad, Silvio Savarese, Richard Socher, Andre Esteva

**Affiliations:** 1 Salesforce AI Research, 575 High St, Palo Alto, CA 94301 USA; 2 Stanford University, 450 Serra Mall, Stanford, CA 94305 USA; 3 University of California, San Francisco, 505 Parnassus Ave, San Francisco, CA 94143 USA; 4 Department of Biomedical Engineering and Physics, Amsterdam University Medical Centers, Meibergdreef 9, 1105 AZ Amsterdam, Netherlands

**Keywords:** Bioinformatics, Image processing, Machine learning, Computer science

## Abstract

Biology has become a prime area for the deployment of deep learning and artificial intelligence (AI), enabled largely by the massive data sets that the field can generate. Key to most AI tasks is the availability of a sufficiently large, labeled data set with which to train AI models. In the context of microscopy, it is easy to generate image data sets containing millions of cells and structures. However, it is challenging to obtain large-scale high-quality annotations for AI models. Here, we present HALS (Human-Augmenting Labeling System), a human-in-the-loop data labeling AI, which begins uninitialized and learns annotations from a human, in real-time. Using a multi-part AI composed of three deep learning models, HALS learns from just a few examples and immediately decreases the workload of the annotator, while increasing the quality of their annotations. Using a highly repetitive use-case—annotating cell types—and running experiments with seven pathologists—experts at the microscopic analysis of biological specimens—we demonstrate a manual work reduction of 90.60%, and an average data-quality boost of 4.34%, measured across four use-cases and two tissue stain types.

## Introduction

The microscopic imaging of tissues, cells, and other relevant biological specimens is key to many areas of biological and medical research. Highly sophisticated tooling and workflows have developed around biological imaging. For instance, molecular staining protocols^1^—chemical stains that selectively highlight different aspects of tissue (e.g., cell types, structures, organoids, etc.)—are used from basic research to medical diagnostics. Furthermore, sample preparation has become highly standardized in a variety of domains (e.g., slide preparation in histopathological analysis^2^), enabling the large-scale digitization of data^3,4^.

Digitization has fueled the advancement of computational methods to analyze data using a variety of techniques. The rise of deep learning methods^5^ in the last decade has spurred progress across most fields that generate sufficiently abundant amounts of digital data. Visual biology is a prime area for the deployment of deep learning-based computer vision (CV) techniques^6^, as evidenced by a rapidly growing body of work^7^. At this intersection of fields, a number of remarkable capabilities have been developed. CV has demonstrated physician-level diagnostic performance on tissue slides^8^, cellular segmentation performance that far surpasses classical techniques^9,10^, the ability to virtually stain raw microscopic images as accurately as chemical stains^11^, and many others.

Supervised learning—in which computational models are trained using data points (e.g., histopathology image; raw microscopy image) and data annotations (e.g., “cancerous” vs “benign”; stained microscopy image)—have been central to the success of CV in biology. Biologists have the distinct advantage of being able to generate massive amounts of data—a single microscopy image can yield a gigabyte of visual data for algorithms to learn from. A disadvantage, however, is the difficulty and cost of obtaining complete annotations for datasets. Consider the ImageNet Large-scale Visual Recognition Challenge (ILSVRC)^6^, a benchmark competition for object classification, localization, and detection in images of normal everyday objects (animals, furniture, etc.). It offered competitors a data set of ~1 million images from 1000 object classes, made possible by the use of crowdsourced annotations from thousands of non-expert individuals. In contrast, computational biology competitions^4,12^ typically offer only hundreds to thousands of labeled examples. The key bottlenecks are that annotators need to have certain levels of expertise and that annotation takes longer than conventional domains, making it difficult to obtain annotations at scale.

Practitioners typically rely on a number of computational advances in deep learning and related disciplines in order to work with smaller annotated data sets. Techniques like data augmentation^10^ can synthetically expand the size of a data set by creating modified copies of the original data that preserve its labels (e.g., distortions, rotations, changes in color balance, etc.), but their key effect is to smooth out data distributions—they cannot synthesize new parts of the distribution. Generative Adversarial Networks (GANs)^13^ have demonstrated remarkable abilities at creating synthetic data, but rely heavily on sufficiently large data sets from which to learn most of a data set’s distribution. Transfer learning, in which models are first trained on large data sets from a different domain (e.g., ImageNet) and then fine-tuned on small data sets for the task at hand (e.g., microscopy data), has become the standard technique across medical and biological CV use-cases^14^. Recently, self-supervised learning techniques—in which synthetic labels are extracted from unlabeled data—have started to mature, demonstrating promise in decreasing the need for abundant labeled data^15^.

In spite of these advances, data annotations continue to be essential in training artificial intelligence (AI), and supervised learning continues to be the standard technique. Significant efforts have been put into developing labeling interfaces that allow experts to efficiently label medical data^16–18^. However, annotating this data for the purposes of AI development continues to require substantial computational knowledge, both in terms of annotating the right data, and training AI models. For instance, variables such as staining inconsistency, scanned artifacts, and natural changes in object appearance, combined with the large amount of data generated in microscopy, can adversely affect the quality of annotated data, and AIs trained from it. Here, the medical field has only just begun exploring AI-based enhancement (e.g., in radiology^19^ and histology^18^).

Here, we present a human-augmenting AI-based labeling system (HALS), in which initially untrained deep learning models learn from human demonstration, train themselves, and begin to augment human annotation ability. The effect is to decrease the user’s overall workload while preserving annotation quality, enabling the annotation of data sets that were previously cost-prohibitive. The system is composed of a set of three different AIs working together and is straightforward to integrate into any image-based labeling tool.

Using challenging and mundane labeling tasks, we demonstrate that HALS can significantly improve the speed of annotation, whereas modestly improving the quality of annotations. Specifically, we outfit a data annotation interface with three deep learning models–a segmentation model, a classifier, and an active learner–which work in synchrony to (1) learn the labels provided by an annotator (2) provide recommendations to that annotator designed to increase their speed, and (3) determine the next best data to label to increase the overall quality of annotations while minimizing total labeling burden. The models work passively in the background without the need for human intervention, essentially enabling a non-computationally savvy biologist to train their own personalized AI for workflow support, and downstream AI development.

To establish an approximate lower bound on human augmentation, we experiment with challenging tasks, working with highly trained expert annotators. Specifically, we select the task of cellular annotation on tissue images–a highly repetitive, time-intensive task, broadly useful across domains of biology–and we select pathologists as annotators. If this method can augment trained specialists on challenging tasks, it is likely to generalize well to less trained annotators on simpler tasks.

We run experiments using four different cellular labeling tasks on two visually distinct stains—Hematoxylin and Eosin (H&E), and immunohistochemistry (IHC). Working with seven pathologists from Stanford and the University of California at San Francisco (UCSF), we demonstrate that our system can reduce the workload of annotators by an average of 90.6%, while slightly increasing the effectiveness of the annotated data by 4.34%. The latter is determined by computing the AUC of accuracy vs a number of training samples for an AI-trained on data annotated with HALS, and comparing it to the AUC of an AI trained without human augmentation. Our contribution is not a new interface, but an AI system that can be integrated into labeling interfaces for human augmentation.

## Results

### System architecture

**Fig. 1:**
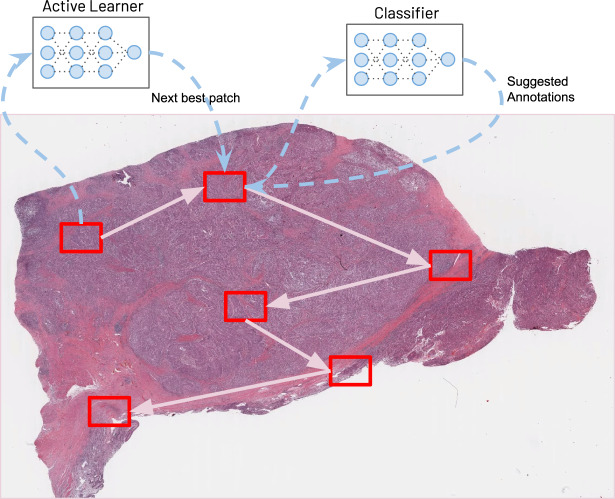
HALS: human-augmenting AI-based labeling system. As a human annotator labels data, an active learning algorithm shuttles the annotator around the image by identifying the next best visual features to annotate. Simultaneously, other AIs make labeling suggestions designed to significantly accelerate annotation speed (not pictured—see Fig. 3. System Architecture). Together, they give annotators the ability to train personalized AI models, enabling them to generate high-quality labeled data sets for otherwise intractably large images. See Data Availability for image details.

The labeling workflow of our system is depicted in the illustration of Fig. 1. Given a large microscopy image (e.g., histopathology whole slide images (WSI), in the provided example), an annotator will begin by labeling points within a small region of the WSI. Once they do, an untrained classifier will begin training itself on these annotations, learning to distinguish between the various classes provided. Once the classifier sees sufficient data (i.e., 10 data points from each class are annotated), it then starts performing two functions. First, it renders suggestions to the annotator, which the annotator may accept or change. In practice, we find that as the classifier’s accuracy improves and the suggestions become indistinguishable from the annotator-provided labels, the speed of annotation significantly accelerates—annotators can scan over a set of suggestions and approve/disapprove much faster than they can individually annotate each point. Second, the classifier converts square image patches that circumscribe the labeled data points into feature vectors which are fed into an active learning model. The active learner takes these feature vectors, along with feature vectors from the circumscribed squares of the remaining cells in the rest of the image, to determine the next best patch for annotation. The net effect of these two models is to essentially guide the annotator around the image, rapidly sampling from a diverse and representative set of points. Along with the rest of the system architecture, they form a human-in-the-loop AI system that learns from demonstration and enhances human performance.

**Fig. 2:**
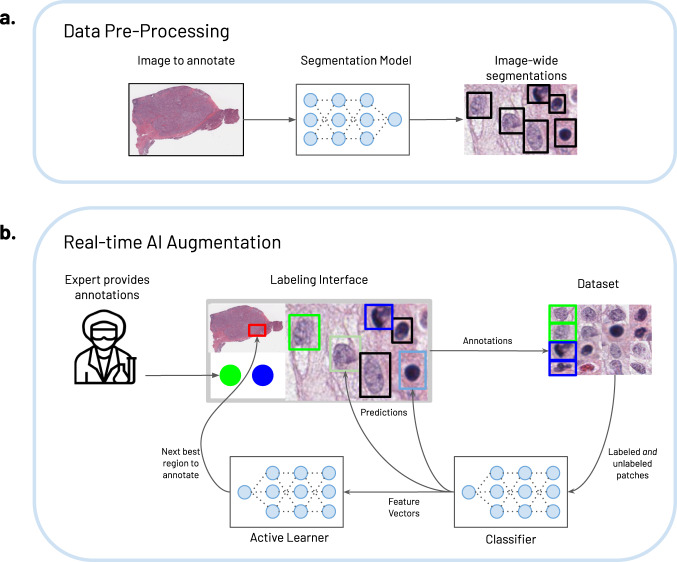
System architecture. **a** Data pre-processing. Digital images are first pre-processed by passing through a deep learning model (HoverNet^9^), which segments and generates bounding boxes for each cell. The image and bounding boxes are then used in real-time via a labeling interface outfitted with two AI models that serve to augment and accelerate expert labeling (**b**). Real-time AI augmentation. As annotators provide cellular labels (bright green/blue boxes denote different classes), they are stored in a data set and iteratively used to finetune a ResNet classification^22^ model, pre-trained on the PanNuke cellular data set, but initially *untrained* on the task at hand. As the classifier learns the annotations, it renders ever more accurate predictions (pastel green/blue boxes) to the annotator. Simultaneously, it feeds in high-dimensional feature vector representations of the labeled and unlabeled data set to the active learner, which determines the next best patch that an annotator should label. Together they increase the speed of annotation and the quality of the labeled data set.

The specific technical steps required to annotate an image break down into two components: (1) a data pre-processing step to prepare the image for enhanced annotation, (2) human annotation through an AI-augmented labeling interface. The structure of this system is depicted in Fig. 2. The first component (Fig. 2a) uses a segmentation model (HoverNet^9^) to segment each cell in the tissue, and determine the smallest containing bounding box for each. In our setup, we train a separate segmentation model for each of the two stains of interest, using the QuPath labeling interface^16^ to generate the requisite cellular bounding box segmentation masks. Given the substantial visual differences between the two stains, we use multiple segmentation models instead of a single multi-task model. Once a segmentation model is trained on a particular stain, it will work for new images that use that particular stain. The positions of these are then sent to the labeling interface for use in real-time. Adapting this step to a new stain type simply requires re-training the segmentation model with an example image.

HALS’s system architecture (Fig. 2b) is built through a microscopy labeling interface outfitted with two deep learning algorithms—a classification model, and an active learning model. We use the SlideRunner open-source labeling interface to build on^20^, a PanNuke dataset^21^ pre-trained ResNet-18^22^ as our classifier, and the Coreset^23^ method for active learning. Both ResNets and Coreset are state-of-the-art models in image classification and active learning, and PanNuke is a data set of 205,343 annotated cells, effective for pretraining this task. Each of these components is completely modular and can be easily replaced with equivalent methods (e.g., a different labeling interface) for new use-cases. Once an annotator begins labeling data points (green and blue bounding boxes, Fig. 2b), the system stores these data points alongside the unlabeled data pool and finetunes the classifier on these labels. Once sufficiently many points are annotated (around 30, in our case), the classifier begins rendering predictions in the interface (muted blue and muted green bounding boxes, Fig. 2b) which the annotator can accept or deny. Further, the classifier performs a feedforward pass over all the data (labeled and unlabeled) and feeds their resultant feature vectors—high-dimensional representations of the cells—into the active learner. This model then determines, from the unlabeled set *U*, an unlabeled subset *S,* which is maximally diverse, and expected to most improve the performance and generalizability of a model trained on *L* + *S*, where *L* is the labeled data. The subset is sent back to the labeling interface, which chooses, as the next regional patch to annotate, the patch that contains the most points of *S*.

### Experiments

To test the impact of HALS on data annotation, we execute two experiments designed to test for improvements to the workload of annotation, and the effectiveness of the annotated data.

#### Annotation workload

Here, an expert annotator is asked to search, within the slide, for a tissue patch that contains ~200 nuclei (~30× magnification), in which they believe the classes are roughly balanced. Each annotator randomly selects a distinct patch. They annotate the patch with AI augmentation as described above, correcting a fraction of the AI’s prediction. The fraction corrected defines their workload. In the limit of workload = 1, this is equivalent to no AI support, in which all annotations are performed by the human. In the limit of workload = 0, this is equivalent to no annotations performed by the annotator.

#### Annotation effectiveness

In this experiment, an expert annotator begins by labeling 20 cells of each class, to initialize the classifier. They then begin labeling cells, following (and possibly correcting) the suggestions of the classifier, while being guided around the slide by the active learner. As a control, they repeat this experiment on the same interface but with all deep learning models deactivated.

**Fig. 3:**
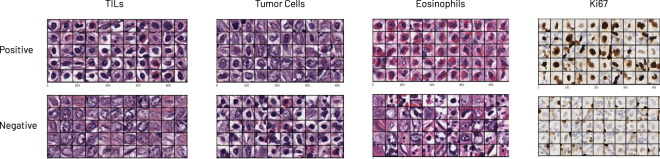
Experimental use-cases. The task of cellular annotation is chosen, specifically owing to its highly repetitive nature, and the difficulty of complete data annotation (e.g., a section of tissue may contain 900,000 cells). Four use-cases highlight the generalizability of this method across stain types and cell types. From left to right: tumor-infiltrating lymphocytes (TILs), tumor cells, eosinophils, and Ki-67-stained cells of arbitrarily sufficient size, as determined by the annotators. Top row shows example cells of the specified type (positive), the bottom row shows examples of all others (negative). See Data Availability for image details.

For each of the experiments, we test seven pathologists (from Stanford and UCSF) on four different binary use-cases (see Fig. 3):

1. **Tumor-infiltrating lymphocytes (TIL) [H&E]**: the presence of sufficiently dense TILs can provide prognostic information and aid in measuring the response to treatments^24^.
2. **Tumor cells [H&E]**: quantifying the fraction of tumor cells in a tissue sample is a challenging task that suffers from pathologist variability, and is of value to therapeutic decision making as well as diagnostics^25^.
3. **Eosinophils [H&E]**: eosinophilic esophagitis is a chronic immune system disease. Quantitating eosinophils is necessary for diagnosis^26^.
4. **Ki-67 [IHC]**: the Ki-67 stain is a marker of cellular proliferation. The ratio of positive to negative tumor cells can have prognostic significance^27^.

In each use-case, the annotator labels two classes of cells: (1) the cell type of interest (2) all other cells in the tissue. All four use-cases are real tasks with diagnostic value. The first three are stained with H&E, while the fourth—stained with IHC—is selectively chosen to demonstrate generalizability across stain types.

**Fig. 4:**
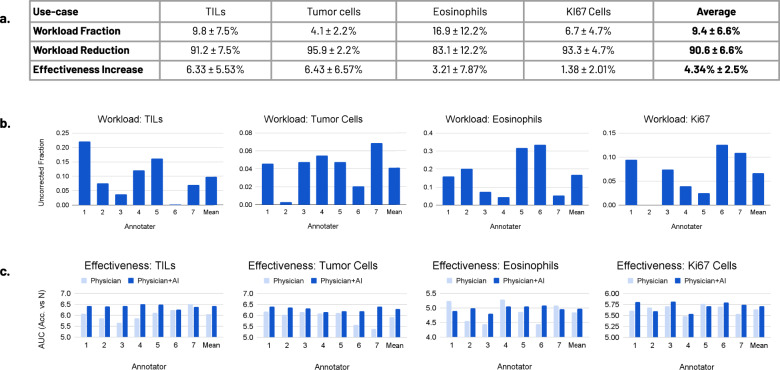
Experimental results. **a** The average workload reduction and effectiveness increase for the four use-cases considered (TILs, tumor cells, eosinophils, and Ki-67 cells), altogether averaging a workload reduction of 90.6% and a 4.34% effectiveness improvement. Workload fraction is measured by the fraction of AI predictions changed by the annotator (0 is perfect model annotation, 1 is without an AI). Workload reduction is the inverse (1–workload fraction). Effectiveness is measured as the area under the accuracy vs number of samples (*N*) plot, bounded by *N* < 200, for an AI model trained on the resultant annotated data set (see example in Fig. 5). **b** Workload results on the seven tested annotators–top histopathology trainees from Stanford and the University of California, San Francisco. **c** Effectiveness results on the same set of trainees.

The results of these experiments are summarized in the table of Fig. 4a. The workload reduction across the pathologists, when using HALS, ranges from 66% to 100%, as measured by their corrected fraction. The average workload reduction is 90.6%. Intuitively, the workload reduction is greater on tasks with greater visual differences between the two classes. Eosinophils (83.1% reduction) are a type of white blood cell with multi-lobulated nuclei and granular eosinophilic cytoplasm, easily confused with red blood cells. In contrast, tumor cells (94.9%), TILs (91.3%), and Ki-67 cells (93.3%) all tend to stand out amongst their respective backgrounds. Individual workloads across the use-cases are shown in Fig. 4b. Some variability can be observed within each use-case. Behaviorally, individual annotators interact differently with the AI, gaining or losing trust in model predictions as a function of model accuracy.

**Fig. 5:**
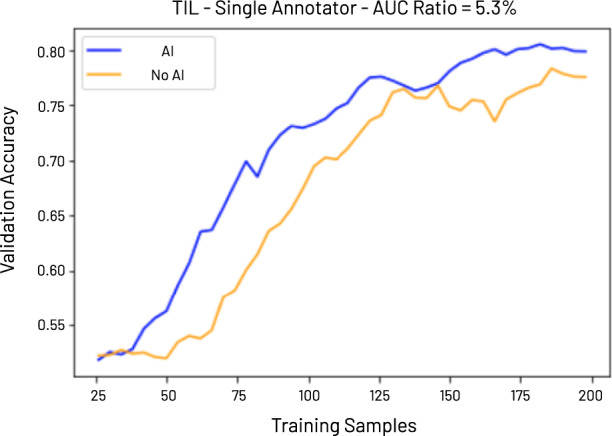
Effectiveness metric. We consider a data set, D, to be more effective than another, E, if a model trained on D has a higher validation accuracy than a model trained on E, and both D and E cost the same to annotate (i.e., both have the same number of data points and annotations). To this end, we use the AUC of a model’s validation accuracy vs number of training samples as a measure of effectiveness, with a higher AUC indicating higher effectiveness and the ratio in AUC between two curves allowing a comparison between two models. In the example above (use-case: tumor-infiltrating lymphocytes), the data set generated with AI augmentation has an absolute validation accuracy improvement of 0.11, 0.11, and 0.05, over a data set generated without AI augmentation, for 50, 75, and 100 training samples, respectively. The AUC ratio of the two curves is 5.3%.

The effectiveness boost across pathologists, when using HALS, ranges from 1.38% to 6.43%, averaging 4.34%. The effectiveness of an annotated data set is defined as the area under the curve (AUC) of validation accuracy versus *N*, the number of training samples, with *N* < 200, for a model trained with this dataset. The AUC of such a curve yields an intuitive measure of how quickly the data set becomes of sufficient quality to learn the task at hand. The higher the AUC, the faster a model converges, the fewer data points are needed to learn the proper distribution. The exact impact of this value on the accuracy improvement of a model trained on the annotated data set is a function of the individual shapes of the AUC curves. See Online Methods for full details on experimental parameters. The effectiveness improvement in one annotated data set over another is then the AUC ratio between them. See Fig. 5 for an example comparison plot of a dataset annotated with HALS versus one annotated without. Here, the AUC ratio is 5.3%, and a model trained with 50, 75, and 100 training examples from HALS benefits from an 11%, 11%, and 5% boost in model validation accuracy, respectively. Given the relatively small data set size, this performance boost is subject to noise. As such, this result serves to show that this method modestly improves data annotation quality while substantially improving annotation workload.

Note that the ResNet classifier used in these experiments is pre-trained on the PanNuke data set—a data set of H&E-stained cells—and observes performance on Ki-67 that is on par with the other use-cases, pointing to the generalizability of this method. Adapting HALS to new use-cases is an iterative process involving testing the system as-is, then potentially replacing the classification and/or the segmentation models with ones trained on the stain at hand. The active learner is generic, operating on feature vectors as opposed to raw input data.

## Discussion

Here we present HALS, designed to learn from human data annotators in real-time, augmenting their abilities and boosting both their annotation speed and annotation effectiveness. It is modularly designed and straightforward to integrate into standard image-labeling interfaces.

Using four highly repetitive binary use-cases across two stain types, and working with expert pathologist annotators, we demonstrate a 90.6% average labeling workload reduction and a 4.34% average improvement in labeling effectiveness. An annotator’s efficiency can be defined as their workload per unit time. In practice, the choice of labeling interface can affect both efficiency and effectiveness. Note that the repetitive work involved in annotation (mouse clicks, the position of UI elements, system lag, etc) can positively or negatively influence annotator efficiency owing to their influence on time. Here, we selectively avoid the challenges implicit in controlling for interface effects by using a time-agnostic metric, the workload, defined as the data fraction corrected. Future work could explore the variation in human enhancement as a function of the chosen labeling interface, tracking the direct impact on time yielded by our method in combination with a given labeling interface. Here, we restrict this work to using the SlideRunner^20^ interface (see Methods), owing to the ease of incorporating modern deep learning frameworks.

Although these experiments focused on binary use-cases, there is no restriction on the number of classes learnable using this method, and future work could explore the effects that increasing the number of classes may have on annotation workload and effectiveness. Further, the classifier in this system can be replaced with a detection or segmentation model, to expand the task type beyond classification. A key limitation to this approach is its dependence on learning accurate object classification in real-time, which requires that slides contain sufficiently many examples of the objects of interest. For large or rare image artifacts, such as entire tumors, additional techniques leveraging few-shot learning may need to be integrated into the system. Further, this technique operates on image regions, and will not help with slide-level annotations.

Decreasing the time and cost of data annotation has the potential to enable the development of previously inaccessible AI models across a range of valuable domains. HALS can serve biologists in data analysis by allowing them to collect quality datasets on their specific use-cases, with minimal computational knowledge, for the training of AI models.

Future work in this direction will involve expanding the capabilities of the system across tasks and image types. This could be achieved by working with more complex biological targets, different stain types, or three-dimensional images (e.g., z-stacks of microscopy cross-sections). Efforts could involve training models that can effectively transfer-learn across similar tasks - for instance, using a single classifier for all cellular annotation tasks, possibly via meta-learning^28^. Eventually, computational layers could be added on top of HALS, which build it into a distributed auto-ML^29^ style platform, which can auto-detect the task at hand, select the best pre-trained model and model type, and concurrently learn from a number of annotators. As this type of technology matures in the research community, decreasing the time and cost of data annotation, more areas of biology will begin to benefit from the high value-add of AI data analysis.

## Methods

This work was reviewed and approved by the Salesforce Research Ethics Team, which offered guidelines for the study procedures. All physician participants consented to be a part of the study.

### Labeling platforms

SlideRunner^20^ is an open-source tool to annotate objects in whole slide images written in Python. The tool supports annotating objects of any size, using point, bounding box, or polygon annotations. It supports plugins that can interact with the data and potentially deploy AI techniques. A version of SlideRunner, augmented with the various AI modules described in Fig. 2, was used for all experiments.

QuPath is an extensive bioimage analysis program written in Java. It contains many built-in workflows for tasks such as cell segmentation and classification. In addition, it is able to process slides in parallel and the program can be extended by writing scripts. QuPath was used to obtain segmentations (i.e., bounding boxes) for the Ki-67 experiments, using the watershed cell detection plugin.

### Segmentation model

Here we use a HoverNet model^9^ for the H&E-stained use-cases, as it achieves state-of-the-art performance against other models (e.g., Unet^10^) at segmentation tasks in tissue. For the IHC-stained use-case (Ki-67), we finetune HoverNet using the segmentation data obtained from QuPath (see above).

### Classification model

Before we can use the ResNet-18 model with a small data set, it needs to be pre-trained. For pretraining, the PanNuke^21^ data set is used, which is composed of 256 × 256 pixel images, containing just over 200,000 nuclei. The images originate from 19 different kinds of tissue and contain 5 different classes of nuclei. All nuclei in the patches have been labeled. We calculated the centroid of every nucleus, and extracted a 40 × 40 patch around the centroid, omitting nuclei at the edges of the images when the window did not fully fit within the image. These small patches are used to train the model, using PanNuke fold 1 and fold 2 for training and fold 3 for testing. Fold 1 and 2 have been combined to calculate the mean and standard deviation in the pixel values. All patches have been normalized by subtracting the mean and dividing by the standard deviation.

To finetune the model, we freeze all layers and replace the final layer with two untrained fully connected layers containing 32 nodes each, with a final output layer containing two nodes corresponding to the binary classification cases used in the experiments.

As the user starts labeling, data are obtained that are used to finetune the ResNet in real-time. The data labeled by the expert is split into a training and validation set. 75% of this data is used for training and the other 25% validation. When the classes are not balanced, the less-common classes are oversampled, in order to present the model with a balanced data set.

The model is optimized using Stochastic Gradient Descent (SGD), with a learning rate of 0.00001 and momentum 0.9. The model is trained for 100 epochs, but is early stopped if the validation score does not improve for 10 epochs.

During system usage, the classification model finetunes itself for every five newly labeled data points (using all labeled data). The cells in the current image patch are predicted and rendered to the user as suggestions. The feature representation for all data (labeled and unlabeled) are then extracted from the second to last layer in the ResNet, and passed to the active learning model to select the next best points to annotate.

### Active learning model

In order to create the best combination of labeled nuclei, we use the active learning method Coreset^23^ to suggest which patch the expert should label next. Active learning methods operate by considering a labeled data pool and an unlabeled data pool, and determining the next best data points from the unlabeled pool to label next. They are optimized to find data points that are highly diverse and will increase both the accuracy and generalizability of the resultant model. Coreset is considered state-of-the-art for image data.

### Workload experiments

At the start of a workload experiment, the annotator is asked to find and annotate 20 objects of each class, to begin the training of the classifier. At this point, the classifier continues to retrain itself for every five additional annotated objects. Once initialized, we ask the annotator to find a patch in the slide that contains ~200 objects that are equally distributed over the classes that are to be labeled and begin annotating the patch. The counts for each annotator and use-case are given in Supplementary Figure 1. During annotation, the system renders predictions to the annotator, which they can confirm or deny until all cells have been annotated to their satisfaction. Once completed, the fraction of annotations corrected is calculated. The fraction corrected defines the workload of the annotator.

### Effectiveness experiments

At the start of an effectiveness experiment, the annotator is asked to find and annotate 20 objects of each class, to begin the training of the classifier, and initialize the active learner’s next-patch suggestion. The annotators then follow the guidance of the active learner until they have annotated 200 total cells. As a control, the experiment is repeated, but with the models disabled.

Owing to the lack of ground truth labels for WSI’s used in the experiments, labeled data across annotators is used as ground truth for evaluation for an experiment. That is, separate classification models (ResNet-18) are trained on a single annotator’s data set, and evaluated on all other annotators’ control data sets. More precisely, if there are N annotators, each with two runs (with and without AI augmentation), then the evaluation of a single run from a single annotator is achieved by training a model on that run’s data and testing it on the N-1 control runs completed by all other annotators. This ensures that the evaluation set is the same for both experiments performed by a single annotator, allowing us to fairly compare model improvements with and without AI-augmented data annotation. The restriction of the evaluation data set to the data used in the control experiment (without AI augmentation) is to eliminate potential biases that may be introduced by an AI, which begins uninitialized and learns as the experiment progresses.

The order in which samples are labeled is preserved during experiments and utilized in generating effectiveness curves (e.g., Figure 5). In such curves, if *N* training samples are used to plot a classifier’s accuracy, those are the first *N* samples to be annotated by the user. We sweep N from 0 to 200 in all curves. In all, 200 is chosen empirically as classifier performance plateaus around this number, in these use-cases. To account for performance fluctuations (particularly for low values of *N*), all models are trained 10 times and the average value is reported.

### Reporting summary

Further information on research design is available in the Nature Research Reporting Summary linked to this article.

## Supplementary information

Supplementary Information

Reporting Summary

## Data Availability

For the TILs and tumor cell use-cases, we used slide TCGA-XF-AAN8-01Z-00-DX1 from The Cancer Genome Atlas^30^ (TCGA). For the Eosinophil use-case, we used slide TCGA-XP-A8T7-01Z-00-DX1 from TCGA. For the Ki-67 use-case we use a slide provided by the University of California at Davis. Not all slides are scanned at the same resolution. Extracting patches around cells of a predefined size in pixels will result in cells appearing scaled. To mitigate this, the patch dimensions are changed such that the rescaled patch always covers the same area.

## Code availability

The code built through this study will be open-sourced and made publicly available through Github. Please contact the authors for further information.
