# Supplementary material for: Biological data annotation via a human-augmenting AI-based labeling system
Source: NPJ Digit Med. 2021 Oct 7;4:145. doi: 10.1038/s41746-021-00520-6 (PMC8497580; doi:10.1038/s41746-021-00520-6)
Supplement: Supplementary file 1 — Supplementary Information [file 41746_2021_520_MOESM1_ESM.pdf]

	TIL			Tumor			Eosinophils			Ki67		
**Annotator**	**P**	**N**	**T**	**P**	**N**	**T**	**P**	**N**	**T**	**P**	**N**	**T**
1	87	104	191	114	74	188	115	98	213	90	117	207
2	92	123	215	86	114	200	88	117	205	91	96	187
3	99	97	196	93	114	207	106	104	210	82	121	203
4	115	92	207	101	91	192	105	105	210	89	121	210
5	109	104	213	96	107	203	112	78	190	108	104	212
6	118	78	196	101	103	204	114	75	189	95	104	199
7	90	123	213	85	128	213	94	99	193	89	110	199

Supplementary Figure 1. Workload Experiment Object Counts. The number of positive (columns “P”), negative (columns “N”), and total (columns “T”) examples for each use-case and annotator during the workload experiments.
