# Supplementary material for: Biological data annotation via a human-augmenting AI-based labeling system
Source: NPJ Digit Med. 2021 Oct 7;4:145. doi: 10.1038/s41746-021-00520-6 (PMC8497580; doi:10.1038/s41746-021-00520-6)
Supplement: Supplementary file 2 — Reporting Summary [file 41746_2021_520_MOESM2_ESM.pdf]

nature research

Corresponding author(s): Andre Esteva

Last updated by author(s): Aug 6, 2021

Reporting Summary

Nature Research wishes to improve the reproducibility of the work that we publish. This form provides structure for consistency and transparency in reporting. For further information on Nature Research policies, see our Editorial Policies and the Editorial Policy Checklist.

Statistics

For all statistical analyses, confirm that the following items are present in the figure legend, table legend, main text, or Methods section.

n/a	Confirmed	
☐	☒	The exact sample size (n) for each experimental group/condition, given as a discrete number and unit of measurement
☒	☐	A statement on whether measurements were taken from distinct samples or whether the same sample was measured repeatedly
☒	☐	The statistical test(s) used AND whether they are one- or two-sided<br>*Only common tests should be described solely by name; describe more complex techniques in the Methods section.*
☒	☐	A description of all covariates tested
☒	☐	A description of any assumptions or corrections, such as tests of normality and adjustment for multiple comparisons
☐	☒	A full description of the statistical parameters including central tendency (e.g. means) or other basic estimates (e.g. regression coefficient) AND variation (e.g. standard deviation) or associated estimates of uncertainty (e.g. confidence intervals)
☒	☐	For null hypothesis testing, the test statistic (e.g. F, t, r) with confidence intervals, effect sizes, degrees of freedom and P value noted<br>*Give P values as exact values whenever suitable.*
☒	☐	For Bayesian analysis, information on the choice of priors and Markov chain Monte Carlo settings
☒	☐	For hierarchical and complex designs, identification of the appropriate level for tests and full reporting of outcomes
☒	☐	Estimates of effect sizes (e.g. Cohen's d, Pearson's r), indicating how they were calculated

Our web collection on statistics for biologists contains articles on many of the points above.

Software and code

Policy information about availability of computer code

Data collection	The SlideRunner open-source data labeling tool was augmented with PyTorch for AI capabilities, and run using Google Remote Desktop for the testing of histopathologists
Data analysis	The Python programming language was used for all analyses.

For manuscripts utilizing custom algorithms or software that are central to the research but not yet described in published literature, software must be made available to editors and reviewers. We strongly encourage code deposition in a community repository (e.g. GitHub). See the Nature Research guidelines for submitting code & software for further information.

Data

Policy information about availability of data

All manuscripts must include a data availability statement. This statement should provide the following information, where applicable:

- Accession codes, unique identifiers, or web links for publicly available datasets
- A list of figures that have associated raw data
- A description of any restrictions on data availability

The microscopy images used in this study are open-source, and listed in the Online Methods section

Field-specific reporting

Please select the one below that is the best fit for your research. If you are not sure, read the appropriate sections before making your selection.

☒ Life sciences ☐ Behavioural & social sciences ☐ Ecological, evolutionary & environmental sciences

For a reference copy of the document with all sections, see nature.com/documents/nr-reporting-summary-flat.pdf

Life sciences study design

All studies must disclose on these points even when the disclosure is negative.

Sample size	7 histopathology annotators were chosen for this study due to the difficulty of obtaining substantially many more. 200 cells were chosen for the efficiency and effectiveness experiments in order to obtain robust measures of cells annotated per second, and model accuracy.
Data exclusions	No data were excluded from the analysis.
Replication	4 use-cases were considered, with 7 experimenters per use-case, for experimental replication.
Randomization	All histopathologists were tested on all use-cases.
Blinding	Blinding was not relevant

Reporting for specific materials, systems and methods

We require information from authors about some types of materials, experimental systems and methods used in many studies. Here, indicate whether each material, system or method listed is relevant to your study. If you are not sure if a list item applies to your research, read the appropriate section before selecting a response.

Materials & experimental systems

n/a	Involved in the study	
☒	☐	Antibodies
☒	☐	Eukaryotic cell lines
☒	☐	Palaeontology and archaeology
☒	☐	Animals and other organisms
☐	☒	Human research participants
☒	☐	Clinical data
☒	☐	Dual use research of concern

Methods

n/a	Involved in the study	
☒	☐	ChIP-seq
☒	☐	Flow cytometry
☒	☐	MRI-based neuroimaging

Human research participants

Policy information about studies involving human research participants

Population characteristics	Seven, early-career histopathologists, from Stanford and UCSF. 4 women, 3 men, all ages 28-34.
Recruitment	Participants were recruited at random from Stanford and UCSF - these institutions were chosen due to prominence in the field of histopathology. The authors intentionally sought out experts in cellular & tissue annotation, for the purposes of the study.
Ethics oversight	This study was reviewed by the Salesforce Research Ethics Team

Note that full information on the approval of the study protocol must also be provided in the manuscript.
